# TML‐6, A Novel Oral Multi‐Target Drug: Design of a Global Phase II Trial Investigating Safety, Tolerability, and Efficacy with Integration of Blood Biomarkers in Early Alzheimer’s Disease.

**DOI:** 10.1002/alz70859_096730

**Published:** 2025-12-25

**Authors:** Chien Hong Lin, Shang Hung Chen, Chia‐Yu Hsu, Hui‐Chen Wang, Ih‐Jen Su

**Affiliations:** ^1^ Merry Life Biomedical Company, Ltd., Tainan City, Taiwan Taiwan; ^2^ National Health Research Institutes, Tainan City, Taiwan Taiwan; ^3^ Southern Taiwan University of Science and Technology, Tainan City, Taiwan Taiwan

## Abstract

**Background:**

TML‐6 is a novel synthetic drug with driving mechanism of amelioration of autophagy function in neurons and microglias to remove intraneuronal and extracellular amyloid accumulation as the potential AD drug. After FDA IND was approved, the phase 1 study in healthy and elderly adults demonstrated good tolerability, no safety issues and ideal dose‐response pk data. Following the phase 1 data, we designed the phase 2 multi‐country trial (US, Sweden, Taiwan) to evaluate benefit of TML‐6 treatment on early‐stage AD.

**Method:**

The global phase 2 randomized and controlled trial was designed to recruit 150 subjects (aged 55 to 85 years) with MCI and mild dementia for TML‐6 treatment, using neuropsychological assessments (CDR‐SB, iADRS) and blood biomarkers (p‐Tau 217, Aβ42/40, NfL, GFAP) as endpoints.

**Result:**

According to the pK data of phase 1, a double therapeutic dose of 400 mg will be used and have no safety issues for Phase 2 trial. Drug efficacy will be analyzed after 12‐month treatment.

**Conclusion:**

TML‐6 exhibits a multi‐target action mechanism by driving autophagolysosomal regulation, meeting the new era strategy to develop AD drug. The combined data of phase 1 trial and chronic toxicology will be applied to US FDA for global phase 2 trial. We look forward to the success of TML‐6 in clinical trials as an novel AD therapy.

**Key Words**:

TML‐6; blood biomarkers; Alzheimer’s disease; autophagy regulation; β‐amyloid clearance; anti‐inflammation

Correspondence to:

Ih‐Jen Su

E‐mail: suihjen0704@stust.edu.tw

